# Continuous Dual Resetting of the Immune Repertoire as a Basic Principle of the Immune System Function

**DOI:** 10.1155/2017/3760238

**Published:** 2017-01-26

**Authors:** Silvana Balzar

**Affiliations:** Department of Clinical Microbiology, Polyclinic Breyer, Zagreb, Croatia

## Abstract

Idiopathic chronic inflammatory conditions (ICIC) such as allergy, asthma, chronic obstructive pulmonary disease, and various autoimmune conditions are a worldwide health problem. Understanding the pathogenesis of ICIC is essential for their successful therapy and prevention. However, efforts are hindered by the lack of comprehensive understanding of the human immune system function. In line with those efforts, described here is a concept of stochastic continuous dual resetting (CDR) of the immune repertoire as a basic principle that governs the function of immunity. The CDR functions as a consequence of system's thermodynamically determined intrinsic tendency to acquire new states of inner equilibrium and equilibrium against the environment. Consequently, immune repertoire undergoes continuous dual (two-way) resetting: against the physiologic continuous changes of self and against the continuously changing environment. The CDR-based dynamic concept of immunity describes mechanisms of self-regulation, tolerance, and immunosenescence, and emphasizes the significance of immune system's compartmentalization in the pathogenesis of ICIC. The CDR concept's relative simplicity and concomitantly documented congruency with empirical, clinical, and experimental data suggest it may represent a plausible theoretical framework to better understand the human immune system function.

## 1. Introduction

Although a remarkable progress has been made in understanding immunity, a comprehensive general model of function of the immune system has not been established. While numerous elements of the immune machinery are identified, the key functional nodes, organizational hierarchy (if any), and how the immune system regulates itself are still not clear.

That is not just a theoretical problem. The lack of basic understanding of immune system's function thwarts efforts toward successful treatments and prevention of steadily growing pandemics of chronic inflammatory conditions such as allergy, asthma, chronic obstructive pulmonary disease (COPD), inflammatory bowel disease (IBD), and autoimmune conditions. According to the World Health Organization's estimate (the global burden of disease, update for 2004), only the chronic respiratory diseases (allergic rhinitis, asthma, and COPD) affect more than 1.2 billion people. Although we have been aware of those idiopathic conditions for decades, their pathogenesis is still unknown. Nevertheless, all are considered immunologic in origin [1, 2].

The current reductionism-driven research has been identifying cellular and molecular mechanisms (such as TNF-*α*, IL-5, and IL-4 pathways) associated with the previously mentioned diseases. However, the subsequently developed immunologicals, which target particular components of the inflammatory cascade, have been somewhat effective in reducing the symptoms, but not in resolving the pathology. Therefore, there is an urgent need to broaden the approaches and find the way to not only therapeutically control those immune disorders, but also develop effective prevention strategy and thus protect the health of future generations. The members of the recently formed trans-NIH Center for Human Immunology, Autoimmunity and Inflammation (CHI) have outlined these problems in the initiative to comprehensively study the human immune system as a pan-NIH project, thus proposing a holistic approach to acquire a deeper understanding of the function of the immune system [1].

In line with those efforts, proposed here is a general concept of function of the immune system. The concept builds on the fact that, due to living system's continuous communication with the environment (energy exchange through heat, food intake, waste excretion, etc.), the system undergoes continuous disturbances and fluctuations in molecular interactions. Therefore, the system is forced to continuously reset itself toward appropriate equilibrium states. Such a continuous equilibrium resetting in the system is a base for the continuous dual (two-way: against self and against the environment) resetting of the immune repertoire (CDR) as a mode of the immune system function.

Description of the CDR-based concept (CDRc) will begin with a brief summary of basic elements and current concepts in immunology as a framework, will include description of the resetting process, and will incorporate those elements to create a general outline of function of the immune system. The last part will focus on compartmentalization of the immune system, which this concept considers essential for establishment and preservation of immune competency. Finally, described is the emerging common mechanism proposed to underlie the pathogenesis of idiopathic chronic inflammatory conditions (ICIC). Concomitant with description of the CDRc are examples of empirically and clinically recognized human conditions, which are interpreted in the CDRc context.

## 2. Methods and Basic Premises

Human physiology and pathology is in focus as the most relevant element in the process of building the CDRc. The approach applies basic principles of living systems' function, current concepts in immunology, and clinical data as the frame and points of reference for this concept.

### 2.1. Living System's Integrity

Living organism actively resists the overall energy drive toward entropy and disintegration into the environment. Immune processes contribute to the maintenance of organism's integrity by sensing/defining its distinction/individuality (self) against other organisms and the environment. That is accomplished through continuous adjustments/resetting of a system to establish a steady state/equilibrium (a) within the system and (b) with/against the environment. One can assume that, to reduce the loss of resources and energy, the surface interactions with the environment and nonself would be energy sparing and simple—preferably nonspecific, passive, and neutralizing (such as protective secretion of mucins, secretory IgA-mediated antigen exclusion, lactoferrin, etc.). Only when those relatively passive protective mechanisms fail are active innate responses triggered, primarily originating from the surface-lining epithelial cells [3]. These basic passive mechanisms are very important, as many elements of self and nonself are of a common origin across all living organisms and thus are likely to have similar molecular structure. Active reactions against those common elements are undesirable, as they carry a risk of cross-reactions and subsequent collateral damage to self. With evolution deriving increasingly complex life forms, the sophistication of self/nonself discrimination mechanisms has been increasing as well. The innate recognition (pattern-recognizing molecules and receptors, PRRs) remains evolutionarily preserved, predominant, and essential even in most complex organisms. Development of additional, specificity-driven adaptive immunity that uses recombination and gene rearrangements to form unique T and B cell receptor (TCR and BCR) specificities has ensured a successful self/nonself discrimination and expansion of jawed vertebrates (fish, amphibians, reptiles, birds, and mammals).

### 2.2. Stochasticity

Randomness of events and uncertainty of outcomes are intrinsic to all processes in nature. Living system can be described as a deterministic chaos of interactions, with outcomes that are predictable only to a certain degree. Evolutionarily successful living organism functions with a degree of (un)certainty that makes its survival and reproduction sufficiently probable.

The complexity of processes governed by randomness of interactions is exemplified in noncovalent molecular interactions typical for immune processes. Those interactions occur due to attraction/repulsion forces between molecules' surface residues (affinity) and are further affected by spatial/structural compatibility (avidity). Those interactions proceed as a continuous process that fluctuates depending on local parameters (temperature, pH, concentration of ligands, etc.). Such multifactorial interactions trigger multiple downstream events whose interactions further expand the range of possible outcomes, all making the linearity in biologic processes highly improbable. As the dynamics and strength of all interactions are influenced by many parameters, outcomes are difficult to predict or control. Obviously, evolutionary selection favored the systems that can function in a stable manner and thus maintain integrity, that is, can successfully compensate for a certain range of instability and fluctuations in a system. The redundancy of system-balancing mechanisms further contributes to the multitude of diverse processes and pathways that assure favorable outcomes—maintenance of integrity at optimal and energy-efficient equilibrium points.* Therefore, living systems are self-regulatory and sufficiently likely to maintain their integrity when functioning within a certain range of parameters.*

### 2.3. Terminology and General Context

Human physiology and pathology is in focus. Further discussion will often refer to conventional T/B cells, whose phenotypes and function are more thoroughly investigated. However, the proposed mechanisms are considered as generally applicable: they may involve different molecules (innate receptors, inter- and intracellular signaling molecules, etc.), but the principle remains the same. Along those lines, immune repertoire includes both innate and adaptive specificities and mechanisms. Term “self” will be used for distinct molecular patterns that are unique to every single living organism (including syngeneic individuals). “Nonself” can be an altered self or a foreign and, in either case, induces recognition—a molecular reaction or signaling [4].

As a consequence of interactions with the environment, living organisms continuously change and, in response to those changes, their* self is also changing*—resetting to reach a new point of inner (within the system) equilibrium, as well as a steady state against the environment. Therefore,* immune reactions reflect a state of a dynamic equilibrium of a system whose likelihood of maintaining integrity against the environment continuously fluctuates.* Those fluctuations could be within or outside the parameters considered physiologic (perception of health versus disease). As the uncertainty of outcomes in such a complex system is intrinsic, learning about the patterns of system's function could identify intervention points with acceptably predictable outcomes, potentially suitable for therapeutic purposes.

### 2.4. Expanding from the Current Concepts in Immunology

#### 2.4.1. Polyspecificity/Cross-Reactivity

The notion is that polyspecificity must be a rule in immune recognition in order for clonal selection to work [5]. Polyspecificity primarily refers to TCR/BCR recognition, while it is certainly applicable to PRRs, chemokine receptors, endogenous ligands' receptors, and so forth. It predicts that, with cross-reactive or degenerate recognition, a smaller number of receptors are required to provide a competent repertoire. Strength of interactions (and thus their likelihood) fluctuates depending on the local parameters, which continuously change the affinity/avidity of interactions. As explained later, that may be crucial for regulation and resolution of the immune response, as well as reestablishment of equilibrium in a system.

Strength of integrated signaling regulates the functional phenotype and survival of cells. It is well established that the T/B cell clonal selection depends on the strength of TCR/BCR signaling from interactions with self [6]. Applied to the peripheral immune setting, along with the notion about integrated signaling, the constellation of various types of signals that cells receive in the periphery (TCR/BCR engagement, costimulatory signals, innate signals, etc.) may determine both the functional phenotype and survival of T/B cells. Indeed, recent reports have shown that integrated innate, endogenous (TLRs, aryl hydrocarbon receptors), and TCR signals are involved in development of a particular T cell phenotype and regulation of adaptive immunity [7–15]. Similar mechanisms have been reported for marginal zone B cells and NK cells as well [16, 17].

In a more general context, the entirety of interactions between different cell populations, including leukocytes and structural cells, and the matrix (through direct interactions, released mediators, exosomes, etc.), contributes to integrated signals that continuously change and regulate the phenotype, function, and fate of all cells [18, 19]. Such interactions modify the system's inner and surface environment and determine the outcomes that maintain system's integrity.

#### 2.4.2. The Self Is Continuously Changing

Normal growth and development, maturation, chronologic aging, various hormonal states (such as pregnancy), and inflammatory and repair processes, as well as the continuous interactions of self with the environment (commensal flora, food intake, etc.) are all associated with (or a consequence of) immunologically recognizable molecular changes of self [20–22]. Those changes, which include permanent or temporary modifications of glycosylation or glycation processes, alter the molecular patterns of self [21–23]. That triggers homeostatic readjustments in the system, including the immune repertoire's resetting independent of exposures to the foreign. Indeed, it has been reported that human marginal zone B cells have molecular footprints of past proliferation and accumulated mutations during fetal life in the absence of germinal centers and independently of antigenic stimulation [24, 25]. Also, memory phenotype T cells have been found before birth in humans and in mice held in germ-free and in antigen-free conditions [13].

The changes of self could be inferred from age-associated differences in the immune repertoire, which is positively selected based on reactivity to self [6]. The limited repertoire for carbohydrate antigens in early childhood (but with already competent repertoire for peptide antigens) suggests that self may be less glycosylated during that early growth/development period and it significantly changes with age [21, 22]. The immune repertoire expands through adolescence, reaches full competence in adulthood, and then gradually decreases to significant reduction in the elderly (reduction in repertoire diversity starts after age of ~50) [26]. Reduction of the repertoire in the elderly affects primarily responses to carbohydrate antigens (which, as it will be explained later, the CDRc considers a consequence of age-related increased glycosylation and glycation and loss of specificities due to the repertoire resetting against self). Related to differences in repertoire and in molecular patterns of self may be the variability in clinical presentation and outcomes of acute infectious diseases in subjects within different age groups. For example, hepatitis A infection in young children is subclinical. However, it causes significant morbidity in adults. Also, pregnancy-associated hormonal environment could induce gestational diabetes. Similarly, metabolic abnormalities caused by diabetes mellitus induce endothelial damage, and so forth. All those observations suggest that self is continuously changing due to both physiologic and pathologic events [21–23, 26–28].

As it will be argued later, the interplay between environment-induced/extrinsic immune reactions and intrinsic changes of self may determine individual's immune status.

## 3. The Immune Repertoire Continuous Dual Resetting- (CDR-) Based Concept of the Immune System Function

The CDRc postulates* continuous dual (two-sided) resetting of the immune repertoire triggered by (1) intrinsic changes of self and (2) interactions with the environment.* Considering the continuity of both processes and constant readjustments to maintain a steady state/equilibrium, the resulting changes in strength of integrated signaling determine the phenotype and survival of cells-effectors and thus the outcomes of immune processes at any given point in time.

### 3.1. Self-Regulation

Polyspecificity and functional plasticity of T cells allow for a TCR-diverse population of activated clones to acquire a range of functional phenotypes (from suppressive to response-amplifying), depending on the strength and constellation of integrated signals that each of the activated clones receives. During immune reaction, a set of activated clones will produce a dynamic curve of responses. On each side of the curve, the extremes of the curve will be clones either deleted or anergized due to the extremes of high or low strength of signaling (resp.) and thus permanently lost or (temporary) silenced. Among the intermediary-reacting clones will be the clones with regulatory function: the low-activated fraction of intermediaries acquiring a suppressive phenotype (IL-10/TGF-*β* realm) and the high-activated fraction of intermediaries acquiring a reaction-amplifying phenotype (Th17 realm). The expansion/contraction of those subsets may fluctuate depending on the strength of integrated signals (local parameters, concentrations of activating self/nonself ligands, endogenous ligands, innate signals, etc.). If the signaling is overall decreasing (due to changed local parameters, ligand neutralization/clearance, downregulation of innate signals, etc.), the activation state of reaction-amplifying and medium-activated subsets will “downgrade” to medium-activated, suppressive, or dormant phenotypes, respectively. Gradually, inflammation will subside and enter the resolution/repair phase, regulating itself according to the changes in relevant parameters. The clones that were not lost in this particular reaction can be activated in a different set of circumstances. Due to polyspecificity, clones can assume roles/functional phenotypes different from their previous experience and become engaged and governed by elements of a new reaction. Therefore,* there are no fixed phenotypes*. Each clone can acquire a range of phenotypes and contribute to self-regulation of different immune reactions. All clones-survivors will be available for activation in another (possibly unrelated, but cross-reactive) response. If activated, they will acquire a phenotype corresponding to the strength of integrated signals at any given point during a particular reaction. It is reasonable to predict that* greater repertoire diversity (and thus higher granularity of specificities) will afford greater regulatory potential.*

It has been observed that the long-term memory in the CD4+ T cell population may not obey strict rules or be as long as previously thought [29]. That observation could be explained by polyspecificity and multifunctionality of clones, which may repeatedly engage in a variety of not obviously related reactions, as well as in response to newly encountered antigens. Similar issues have been recently reviewed as related to vaccination responses in the elderly [30]. Also, recent report suggests that long-term antibody responses are maintained in part by long-lived plasma cells, but 40–50% of plasma cells are newly formed cells from clonally disparate precursors [31].

### 3.2. The Resetting Process in Tolerance and Repertoire Fluctuations

As described, adaptive immune response will engage a population of T cells consisting of clones with a range of TCR specificities, which will behave as* a dynamic entity that keeps changing its phenotype profile* and adapting in response to a dynamic constellation of parameters, adjusting cells' function/phenotype accordingly. At any point during such a process, additional dormant clones can be engaged, and overly activated clones can be lost.* Such a loss of clones/specificities resulting from an adaptive reaction will effectively reset the overall repertoire.*

The observed sepsis-induced T cell loss and alterations within the CD4 T cell compartment are an example of repertoire resetting and attrition after a violent systemic adaptive response. Postsepsis alterations include reduced repertoire diversity, changed CD4 T cell subpopulation profiles, and suppressed immune function, with increased susceptibility to secondary infections [32].

Simultaneously with the resetting during an adaptive response similar repertoire resetting occurs as a homeostatic mechanism of adaptation to the changes of self. As mentioned before, those changes of self are intrinsic to growth, maturation, and aging but also are induced by stress, inflammation (injury, infection), pregnancy, metabolic abnormalities, and so forth. As with adaptive reactions, the changes of self can engage dormant cells that have been minimally self-reactive, already active clones can reach a state of inappropriate activation, and additional signaling input can eliminate those in a state of strong activation. In addition, dormant clones can die out of neglect if the newly changed self no longer provides the positive reinforcement signals. In this context,* tolerance reflects a dynamic state of homeostasis maintained by a successful resetting of the immune repertoire against both self and nonself.*

One can appreciate how continuous resetting of the repertoire can both expand (engagement of dormant and/or naïve cells) and reduce (deletion due to hyperactivation) the available range of specificities.

In parallel, the innate repertoire (adhesion molecules, PRRs, innate receptors, etc.) is continuously changing through the degree of expression, tachyphylaxis, due to secondary molecular modifications (changes in glycosylation, glycation), and so forth, thus affecting the strength/quality of interactions and integrated signaling.

It is important to note that* the resetting processes will be more pronounced with more frequent activations of adaptive responses*. Adaptive reactions will keep inducing the resetting of the repertoire in addition to its continuous homeostatic resetting against self. It should be also kept in mind that the expansion of the T cell repertoire is limited by availability of naïve clones and involution of the thymus. In addition, due to glycosylation/glycation or other changes of self, the B cell repertoire may shift/decline with age, with altered repertoire particularly against the carbohydrate epitopes. Therefore, there is a breaking point when an overall immune decline starts. From that point on, any future immune interactions (homeostatic or adaptive) are likely to result in* repertoire attrition.* Consequently, due to the reduced repertoire's granularity, the regulatory potential and the tolerance range will decrease as well.

Taken together,* the CDRc allows for regulatory mechanisms to emerge at all times and in response to a range of nonself- or self-induced challenges. It predicts acquisition and maintenance of tolerance as a continuous and dynamic process, which stems from and depends on the available immune repertoire.* Owing to the redundancy of mechanisms and polyspecificity/degeneracy of interactions, the cells' functional plasticity and resetting processes may preserve the competency of the system and successfully compensate for repertoire attrition (intrinsic to this concept), with relatively minor disturbances in the system. Those could manifest as subclinical inflammatory responses and allergic reactions. Significant alterations/reductions of such a redundant repertoire can normally be expected with advanced age (involution of thymus, progressive alterations of self), but also as a consequence of violent or persistent adaptive responses (extensive injury, sepsis, perpetual infections, and chronic inflammatory syndromes). Those can gradually reduce the repertoire's diversity and its regulatory potential and start engaging less appropriate mechanisms. Such a sequence of events may increase probability of unfavorable outcomes, cause instability in the system, and eventually lead to the irreparable loss of system's integrity—death.

## 4. Carbohydrate Antigens, Glycosylation, and Immune Repertoire Resetting Associated with Age(ing)

Carbohydrates and their moieties are ubiquitous in nature. Virtually all living organisms (prokaryotes and eukaryotes, plants, fungi, and animals) are coated with carbohydrates or carbohydrate residues. In mammals, glycosylation is a part of posttranslational protein modification and lipid alterations, providing their structural stability and defining interactions with other molecules [33, 34]. Glycosylation is differentially regulated during development, growth, and maturation. In addition, protein-glycan interactions are recognized as pivotal in controlling innate and adaptive immune responses [35–37].

**Figure 1:**
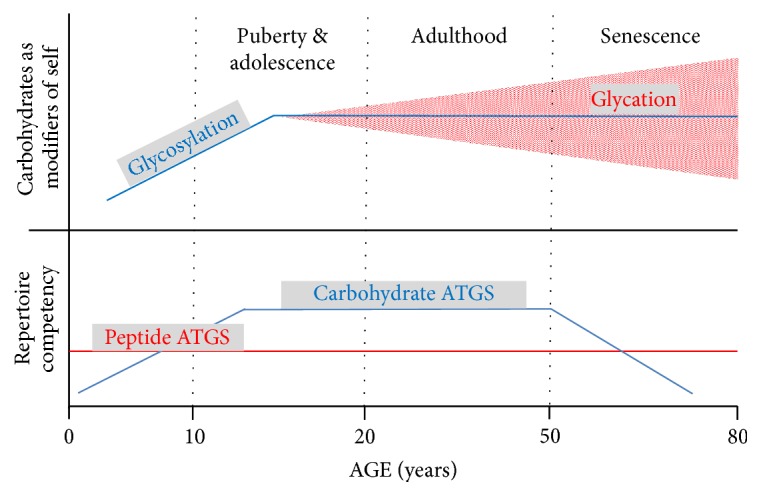
Concomitance of the age-related changes in glycosylation and glycation patterns with the age-related repertoire competency expansion and contraction/attrition. Competency for peptide antigens develops early in life and persists throughout life. In contrast, repertoire for carbohydrate antigens develops gradually. It parallels the growth- and maturation-induced glycosylation of self (first ~15 years of life), to which the prominently active thymus during that period of life responds with an output of carbohydrate-responsive repertoire. That repertoire contracts later in life, paralleling increased alterations of self due to potentially pathogenic glycosylation and/or glycation processes. Those alterations may be primarily induced by environmental factors (injury, infection, nutrition-induced metabolic abnormalities, etc.) and may result in repertoire attrition.

Such ubiquity of carbohydrates in nature and their involvement in virtually all biologic reactions make the cross-reactivity in the process of self/nonself discrimination intrinsic and unavoidable. Therefore, immune reactions to carbohydrate antigens present a delicate task for the immune system [38]. Indeed, while responses to peptide antigens are well developed even in very young children, the repertoire of responses to carbohydrate antigens develops gradually and may reflect a dynamic balance between the changes in glycosylation patterns of a growing/developing self and interactions with the sugar-coated environment (Figure 1). The immune competence of healthy individuals steadily increases during childhood (e.g., children remain susceptible to* Streptococcus* spp. and* Haemophilus* sp. infections up to adolescence), remains at similar levels by ~50 yrs. of age, and then declines [26, 30]. The slow development of carbohydrate-responsive repertoire coincides with growth and maturation, the delay likely allowing for appropriate glycosylation of self to proceed (Figure 1). The growth/maturation-associated glycosylation gradually expands the carbohydrate “self template” for carbohydrate-responsive repertoire expansion. The increased susceptibility to bacterial infections again later in life suggests that aging may include changed and/or increased glycosylation/glycation of self [21–23]. According to the CDRc, the continuous resetting processes will deplete the repertoire becoming overly reactive/cross-reactive with the glycosylated/glycated self. Consistent with the concept is also homeostatic (against the altered self) activation of previously dormant and low-reactive clones, which may become intermediary self-reactive. That would result in reduced repertoire of conventional T/B cells, particularly to carbohydrate antigens (leading to increased susceptibility to infections), and also inception of chronic inflammatory conditions (increased self-reactivity coupled with progressively limited regulatory potential). In addition, due to alterations in glycosylation/glycation patterns of self, innate immune environment will change as well, including the expression of PRRs, reportedly altered in the elderly [27]. Therefore, the mechanisms to maintain integrity/distinction against the environment will become less granular (due to the overall repertoire reduction) and less specific. Progressive attrition of the repertoire may be setting different homeostatic points for the immune system in the elderly as compared to younger adults, with likely increased baseline inflammatory state—noted as “inflammaging” [39]. The self is becoming more similar to the environment and progressively less competent to distinguish between itself and nonself/environment or to maintain the integrity of the system, which eventually results in death. Therefore,* glycosylation/glycation processes may regulate not only growth and development, but also aging and longevity. *The changes expected per CDRc are consistent with the observed immune remodeling/immunosenescence associated with both, chronic inflammatory conditions and aging [26–28, 30, 32].

## 5. Compartmentalization of the Immune System: A Necessity

Recognition and neutralization of a foreign without harming the self are formidable tasks considering the common origin and inevitable molecular similarities among all living organisms. In the course of evolution, as the complexity of organisms increased, the ever more sophisticated immune mechanisms to achieve high specificity of recognition (epitomized in function of T and B lymphocytes) represent an important evolutionary survival tool. But it is also a dangerous one, as reacting indiscriminately and specifically to everything that is not self is unsustainable. This is where compartmentalization (demonstrated to function in mammals), with the mucosal immune compartment as a barrier between the environment and the systemic compartment (representing a sequestered and isolated* genuine self*), becomes an essential survival strategy in highly organized megaorganisms [40].

The surface-lining mucosal compartment uses a versatile set of mechanisms to achieve a low-maintenance homeostasis at the interface between the self and the foreign/environment. Functioning “on the edge,” it locally deals with not only potentially invading entities, but also innocuous antigens—all of which may have a certain degree of similarity with the self. In order to minimize inflammation, mucosal compartment may favor the versatility and general applicability of innate mechanisms over the conventional adaptive immune responses as a practical approach to deal with the environment. More of a foreign may be “allowed” at mucosal surfaces, without inducing major immune reactions or engaging conventional adaptive immune responses. To accomplish such a delicate task, mucosal compartment is equipped with a unique repertoire of unconventional effectors and mechanisms. Those ultimately define the system's capacity to deal with the environment and maintain integrity in the context of self. Along with epithelial cells and their particular capacity to recruit innate effectors, unique populations of evolutionary old cells reside along mucosal surfaces and are seldom found elsewhere. Those resident populations include subsets of unconventional T cells, mucosal B cells, and mast cells. The adjective “unconventional” applies to both mucosal T and B cells, as their repertoire (T-independent antigens such as carbohydrates and lipids) and efficient activation process (polyclonal/T cell-independent or neutrophil-mediated B cell activation, dispensable “classical” costimulatory signals for T cell activation) enables them to develop a unique repertoire and, having prestored mediators, react without delay [41–46]. Mast cells, residing in such a setting of continuous self/nonself interactions, may be essential for regulation of responses necessary to maintain a sustainable equilibrium with the environment [47, 48].

Thus, mucosal compartment can be considered a site of a particular immune privilege, where direct interactions with nonself are “allowed” and tolerated at the level not desirable (nor sustainable) within the systemic compartment. Consequently, mucosal compartment's repertoire must differ from the systemic repertoire. Considering the continuous resetting processes, the CDRc requires that the mucosal compartment should remain relatively isolated from the systemic, genuine self. That would enable the mucosal compartment to avoid significant resetting against the systemic/genuine self (and vice versa) and reset only in response to local changes of self resulting from interactions with the environment. That makes the mucosal self different from the sequestered and protected genuine self. The sequestration from the systemic compartment protects mucosal compartment's unique repertoire and preserves its specificities accumulated from early life on.

Accordingly, frequent failures of the epithelial barrier function will induce resetting of the mucosal repertoire and alter the mucosal compartment's immune environment. Allergic reactions may indicate such an altered mucosal compartment and reflect activation of alternative mechanisms to maintain equilibrium along the body surfaces, but at a less appropriate (i.e., symptomatic) level.

The mucosal compartment's repertoire (including innate and adaptive repertoire of mucosal B and T cells) may develop and expand primarily during early childhood, as a result of interactions with the environment, and continue expanding through adolescence. Only when growth and development (i.e., growth-induced alterations of self) are finalized, does the mucosal compartment achieve its maximum competency.

Previous comments about the age-associated development of immune repertoire against respiratory pathogens support that notion. Also, children with asthma-like respiratory symptoms sometimes “outgrow” those by adolescence/adulthood, presumably owing to the maturation and increased competency of the mucosal compartment.

Therefore, the maturity and competency of the mucosal compartment acquired early in life determine how often the systemic exposure to the foreign may occur and, if it occurs, to which extent the foreign can be preprocessed/neutralized before breaking through the mucosal barrier. On such occasions, the intrinsically self-damaging activation of the systemic adaptive responses (conventional systemic repertoire is selected and maintained due to its low self-reactivity) will trigger the resetting process. The resetting will occur due to both, reactions against the intruding foreign and against the reaction-altered genuine self. As both processes can engage dormant repertoire, as well as reduce the activated repertoire, high frequency of such events may increase the probability of significant depletion of the overall repertoire, reduce its regulatory range (reduced granularity), and thus result in inception of chronic (intrinsically autoreactive) processes.

**Figure 2:**
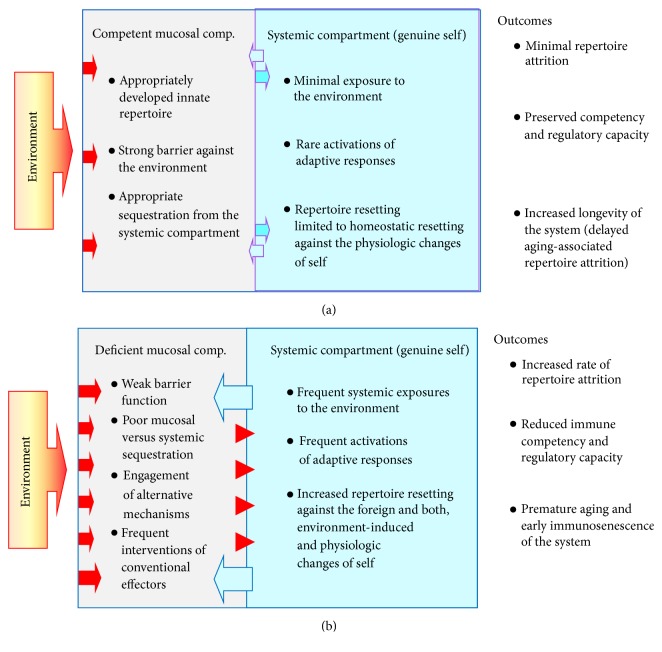
Compartmentalization: competent mucosal immune compartment is essential for appropriate function of the immune system. The CDR concept indicates favorable outcomes in a system with strong mucosal barrier function and appropriately sequestered systemic immune compartment (panel (a)). Panel (b) shows unfavorable outcomes in a system with deficient mucosal barrier function and poorly sequestered systemic immune compartment.

Consequently, the CDRc indicates that the “prime directive” of immune protection is to isolate the systemic compartment (genuine self) from exposures to the foreign. In that way, the systemic repertoire will undergo only a homeostatic resetting due to the intrinsic changes of self and avoid additional resetting events against both the intruding environment and the alterations of self due to such intrusions. Such a state of systemic compartment's isolation may preserve the systemic repertoire and its diversity, provide appropriate regulatory breadth/capacity, and thus delay immunosenescence (Figure 2(a)).

Although immunosenescence usually occurs with advanced age (also referred to as age-associated immune remodeling), immunosenescence occurs also in younger subjects as a consequence of major pathologic events or associated with chronic inflammatory conditions [26, 32]. Related to an altered mucosal compartment is depleted and phenotypically changed mast cell population, as well as a form of immunodeficiency affecting responses to carbohydrate antigens that are both observed in severe asthmatics regardless of their age [48, 49].

Therefore, the CDRc indicates* that appropriately developed and preserved mucosal immune compartment is essential for optimal function and regulation of immune responses in general.* Strong and competent mucosal barrier ensures individual's immunologic fitness and delays immunosenescence, despite the chronologic aging. As Figure 2(b) depicts, reduced immune competency along mucosal surfaces can trigger a sequence of alterations in the entire immune system, which can eventually reduce the overall repertoire (repertoire attrition), impair the protective function and regulation of immune reactions (due to reduced repertoire's granularity), and force the system to continue acquiring pathologic steady states. Such a sequence of events, which reduces self-regulatory capacity of the immune system, may be involved in the pathogenesis of chronic inflammatory conditions and autoimmune diseases. Allergic reactions in this context may reflect engagement of alternative mechanisms (eosinophils, increased IgE, increased mast cell counts and activity, etc.) to cope with otherwise innocuous environmental exposures. That would indicate an altered competency of the mucosal compartment and its barrier function and thus can represent a prodromal stage of a progressive and potentially detrimental immune deficiency.

## 6. Pathogenesis of Idiopathic Chronic Inflammatory Conditions (ICIC)

The CDRc suggests a common mechanism related to the altered mucosal protection underlies the pathogenesis of ICIC (Figure 2(b)). Under such circumstances, the stochastic nature of immune processes may produce a range of outcomes, resulting in variable clinical presentations. Indeed, ICIC are highly heterogeneous syndromes that, rather than having pathognomonic features, are clinically defined using sets of major and minor criteria. The overlap between various syndromes is very common.

For example, interstitial lung disease symptoms often precede rheumatoid arthritis. Also, there can be a considerable overlap between asthma and COPD. Despite the obvious differences in lung pathology between those two syndromes, some asthmatics demonstrate fixed airflow obstruction or a Th1 inflammatory pattern typical for COPD, while some COPD patients have partially reversible airflow obstruction and a Th2 inflammatory pattern typical for asthma. Nevertheless, altered lung mucosal immunity underlies both diseases.

That suggests that various triggering mechanisms and diverse pathways resulting in altered mucosal protection could lead toward similar pathology and clinical presentations. As there is currently no way to predict which circumstances or events would (or not) trigger an ICIC, the prevention strategy should be quite general and comprehensive: to support appropriate development of the mucosal compartment and to maintain its barrier function and competency. On the other hand, the therapy should primarily identify and reduce/block the mucosal triggers that cause worsening of the disease. Those may be different in each patient, which means that therapeutic interventions will depend on the immune status and circumstances specific for each patient. Therefore, in order to both prevent and start treating ICIC, immediate research focus should be on finding the means to reinforce mucosal barrier function. Future research may lead toward understanding the specific pathogenic mechanisms and identification of specific therapeutic targets to control the pathology.

**Figure 3:**
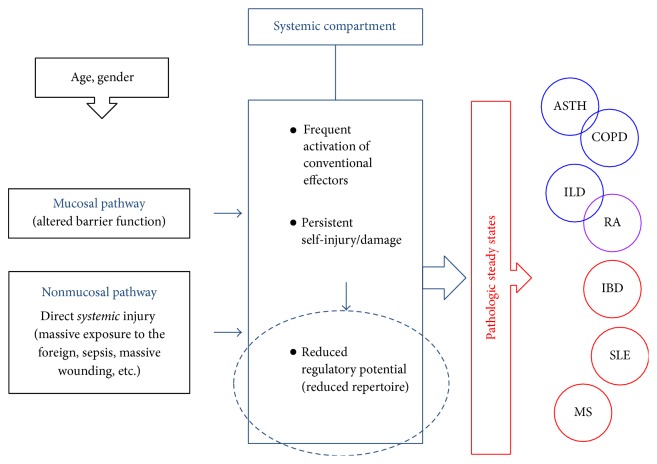
Pathogenesis of idiopathic chronic inflammatory conditions (ICIC). Shown are some of the factors that can affect the systemic immune compartment and contribute to the development of ICIC (only few are listed). Note the reduced regulatory function of the systemic compartment as a common mechanism in pathogenesis of ICIC. Also, note the influence of age and gender. ASTH = asthma; COPD = chronic obstructive pulmonary disease; ILD = interstitial lung disease; RA = rheumatoid arthritis; IBD = inflammatory bowel disease; SLE = systemic lupus erythematosus; MS = multiple sclerosis.

Figure 3 expands on the mechanisms of ICIC pathogenesis proposed in Figure 2(b). It includes also the previously mentioned direct exposures of the systemic compartment. Also, added are the influence of age at inception of pathologic processes and the role of gender. Generally, women are more likely to develop ICIC, which could be related to their typically Th2-dominated immune environment. Of note, mucosal immune responses also have a strong humoral/Th2 immune component. Those elements suggest the role of B cells in development of ICIC in a setting of reduced T cell regulatory potential. Noteworthily, the B cell repertoire (unlike with T cells) continues to be replenished throughout life but influenced and limited by the changes of self. Depletion of B cells with rituximab and similar biologicals, which is in fact a form of B cell repertoire resetting, has been reported as an effective intervention for several autoimmune diseases.

## 7. Discussion

This study proposes the CDR as a mechanism that underlies a comprehensive picture of the human immune system function. Referencing and relying primarily on clinical and experimental data, the study outlines a dynamic, continuously changing, and self-regulating biologic system governed by stochastic events and uncertainty of outcomes. When set in motion, the CDRc explains a range of immunologically distinct physiologic and pathologic phenomena: compartmentalization, physiologic growth and maturation-associated repertoire development, immunologic abnormalities, and repertoire alterations associated with idiopathic chronic inflammatory conditions and premature immunosenescence, as well as aging-associated immunologic changes (i.e., chronologic immunosenescence). Finally, the CDRc successfully tackles the unresolved issue of tolerance and regulation of immune responses [50]. It incorporates fundamental principles of thermodynamics and the stochastic nature of biologic systems' function to describe dynamic regulation and maintenance of tolerance. The basic principle of CDR is relatively simple, has internal logic and consistency, and incorporates much of the known in immunology.

Many of the pertinent elements of the CDRc are based on our current understanding of immunity. In fact, the CDRc utilizes functional plasticity of cells and proposes that fluctuating constellation of signals allows any activated cell to change its functional phenotype accordingly. The plasticity of T cells (but also NK cells, B cells, mast cells, fibroblasts, epithelial cells, etc.) by modifications of their stimulatory environment has been demonstrated in numerous recent studies [7–12]. Those experimental data were crucial in explaining the dynamic nature of tolerance and regulation of immune responses.

The CDRc expands primarily from elements of the Danger Theory and continues to use the concept of self-nonself [51]. In contrast to predominant views, conventional adaptive immune responses are not considered the main mechanism of immune protection. The CDRc instead emphasizes the importance of innate mechanisms, while the true conventional adaptive effectors are kept secluded within the systemic compartment and, preferably, seldom engaged. The lack of innate memory could be an argument against the reliance on the innate. However, the dogma about innate immunity that, as opposed to adaptive immunity, cannot provide memory responses has been challenged. Recent reports on trained immunity convincingly argue for the existence of innate memory. It functions in plants, invertebrates, and vertebrates and can provide both specific and cross-protection, as well as engage in alloreactions [52, 53]. Indeed, innate recognition of allogeneic nonself is reported as a base for alloreactivity in MHC-matched murine transplant models [4]. That argues also for an innate molecular imprint of self, a plausible mechanism for “self-awareness” under the CDR conditions and continuous changes of self.

The notion about self has been present in immunology for decades. The self is considered an immunologically constant and unique quality that remains unchanged throughout individual's life. However, the definition and what constitutes self remain unresolved [54]. The CDRc proposes a different perception of self. The self per CDRc is a state of molecular congruency/compatibility that is derived from system's integrity/existence and keeps a system in homeostasis. Particular molecular states and patterns, which are subjected to continuous resettings, constitute the system's selfness. As the self continuously changes, it cannot be precisely defined, nor does the immune system have the ability to specifically identify it—it can only sense a change and reset against it.

As emphasized before, the protection of system's integrity relies primarily on innate mechanisms. However, what makes the integrity-sustaining resetting processes personal is the MHC context. The system uses adaptive effectors to compensate for disturbances whose characteristics and/or extent reach a threshold for triggering an MHC-focused/guided response. Specificity-driven adaptive response (as opposed to less restricted innate reactions) has the ability to, mainly through innate effectors, neutralize (eliminate or tolerate) and repair the damage, that is, to actively regulate inflammatory processes in the context of integrity of the system. MHC-guided responses are to limit the collateral damage by being specific (and also self-focused, determined by self) and to restrain/regulate inflammatory reaction as much as the immune repertoire's diversity affords it. Thus, specific reactions to nonself/foreign are balanced and regulated as relative to self. They affect both nonself/foreign and self (damage it and/or modify it). Consequently, self is included in the system's resetting processes and is (apart from its genetically determined core structure) continuously modified. As long as the system can reset and establish a sustainable steady state, a changed self will become a new self. In this context, an MHC-focused adaptive immunity constitutes a sophisticated mechanism of regulation toward integrity protection in highly organized biologic megasystems.

While the system recognizes the nonself/foreign as a change/disturbance at molecular level, there is no inherent and obligatory animosity (attacking and eliminating at all cost) against nonself/foreign. As the prime purpose of immune responses is to preserve the integrity of the system, the system can acquire even pathologic steady states if that is the thermodynamically optimal solution that will maintain system's integrity. Therefore, reactions to nonself/foreign can eliminate it or, if thermodynamically preferable, those processes could result in asymptomatic/subclinical or chronic symptomatic states (parasite or virus carriage, ICIC, etc.).

As previously emphasized, the fundamentals of system's integrity are as follows: (a) appropriate surface protection with sustainable equilibrium with the environment; (b) system's inner equilibrium. From those elements stem compartmentalization and consequences of failures in either of those areas (Figures 2 and 3). As a consequence of compartmentalization, the CDRc distinguishes between the mucosal self and the systemic, genuine self. Genuine self is hidden within the systemic compartment and exposed to the conventional immune repertoire. When protected by a barrier of the mucosal compartment, extrinsically induced changes of the genuine self are minimal and fluctuations in the systemic (conventional) immune repertoire are mostly due to intrinsic/homeostatic resettings only. As shown in Figure 2(a), that preserves the stability and longevity of the system. In contrast, altered barrier function of the mucosal compartment exposes the genuine self to changes due to engagements of the conventional immunity, which eventually leads to repertoire attrition and reduces the immune repertoire's granularity and thus its regulatory function. That may explain the observed association of the systemic inflammatory conditions and autoimmune diseases with an altered mucosal immunity. Although clinical presentation and outcomes of those conditions may differ depending on the degree and segments of immunity affected, the common mechanism of pathogenesis suggests that directing research toward finding a way to support the mucosal compartment's competency development and its barrier function may lead toward effective prevention and therapy for a range of pathologic conditions. Similar approaches may prevent a disease-induced immunosenescence, as well as delay the physiologic aging and the aging-induced immunosenescence. In the context of the CDRc, interventions and therapy approaches to inhibit or eliminate particular elements of the inflammatory cascade would be contraproductive.

The CDRc indicates that skipping the mucosal compartment and introducing antigens to the systemic compartment directly (such as immunization via parenteral route) may result in harsh alterations of the systemic compartment's conventional repertoire and the molecular structure of genuine self. Because of the potentially detrimental impact particularly during early childhood, a period of organ development, growth, and maturation (appropriate molecular interactions are of paramount importance in those processes!), the changes in immunization strategies would be necessary. The immunization strategy should favor a nonaggressive antigen introduction via mucosal surfaces, particularly for infectious agents that use mucosal route of entrance. In addition, the timing of interventions to modify the competency of the mucosal compartment may have to be synchronized with child's age/maturation state. Of note, there is a potential to exploit the cross-reactivity and perhaps look for “multifunctional” molecular patterns instead of multiple microorganism-specific antigens, which may provide a wide range of cross-protection.

There may be implications for transplant immunology as well. The stochastic nature of a process determining the continuously changing “selfness” of every single individual explains why even syngeneic individuals are immunologically different. In addition, autologous tissues obtained early (or earlier) in life and preserved for potential use later in life may not be as compatible as expected. Furthermore, malignancies indeed stand a good chance of remaining unharmed by immune reactions, as long as the system resets itself against the rogue malignant cells (and vice versa) within acceptable parameters. In fact, the changes in intercellular communication/signaling resulting in uncontrollable proliferation (as a consequence of initial injury/irritation) could have been part of resetting processes to adjust and establish thermodynamically optimal state(s).

Studies designed by teams of experts from various fields of natural sciences will be required to validate the CDRc. The currently available methodologies, particularly recent advances in the field of glycobiology, should provide at least initially sufficient investigative tools. Data analyses should include mathematical modeling approaches. Of crucial importance would be to demonstrate physiologic (and pathology-associated) patterns of changes of self. Therefore, the hypothesis is that individual's self is changing throughout life due to the growth and maturation processes, physiologic and pathologic events, and aging. Those changes will be gender-distinctive. The changes will associate with predictably different patterns of immune markers that will correspond to the changing states of self.

Experimental approaches to validate the CDRc should include prospective longitudinal studies of humans, with sampling at the age-related and other immunologically distinct points. Similar but more detailed studies in animal models and/or in model organisms can be used for mechanistic studies as well. The areas of interest are as follows:

1. Glycosylation and glycation (G/G) processes associated with (a) physiologic development, growth, maturation, hormonally distinct periods of life (adolescence, pregnancy, and menopause), and aging; (b) acute systemic reactions (sepsis, significant systemic injury/wounding); (c) chronic inflammatory conditions. Such studies will address the proposed continuous physiologic and pathologic changes of self.
2. Longitudinal assessment of changes in the systemic/serum immunoglobulin (Ig) response patterns (Ig classes, IgG and IgA subclasses, and their glycosylation characteristics) to a standardized set of T-dependent and T-independent antigens. Those studies (concomitant with G/G sampling) will address the proposed changes in immune repertoire's diversity associated with the previously outlined physiologic and pathologic states.
3. The relationship of innate and adaptive cellular and humoral markers (changes in their molecular patterns and characteristics) to the previously outlined physiologic and pathologic states and other concomitantly evaluated elements.

In conclusion, this study offers a testable theoretical scaffold for translational research based on systems biology approaches. It crystalizes a single CDR principle to outline a relatively simple and somewhat dispassionate view of function of the immune system: it is all about continuous molecular rearrangements and resettings to maintain system's integrity, without the drama of a battlefield and warfare against the unknowns. Dysregulation of immune reactions, which is often considered a cause of various idiopathies, may not be applicable in its true sense: self-regulation is intrinsic in a living system, as molecular interactions are always thermodynamically optimal under any given circumstance. Although it is clear that certain types of cells are specialized immune cells, every single cell, a single entity itself, is involved in maintenance of megaorganism's integrity and thus is part of the immune system.

## 8. Conclusions

This study, initiated to clarify the pathogenesis of chronic inflammatory conditions, confronts the most pertinent question in immunology: general picture of the immune system function. The studies have crystalized the continuous dual resetting of the immune repertoire (CDR) as a basic principle of the immune system function.

The CDR process maintains continuous fluctuations in the immune repertoire diversity, which plays a major role in regulation of immune responses, tolerance, and immune repertoire's physiologic changes from early childhood to senescence. The continuous repertoire resetting inevitably leads to the repertoire attrition. That results in a form of immune deficiency, with reduced repertoire's granularity and immune system's regulatory capacity. Such repertoire attrition is normally expected with advanced age (aging-associated physiologic immunosenescence). However, pathologic events can trigger a premature form of such immune deficiency, which can result in altered immune responses, chronic inflammatory conditions, and autoimmune diseases.

Following the consequences of the CDR-induced repertoire attrition, proposed is a common mechanism of pathogenesis of idiopathic chronic inflammatory conditions. Their pathogenesis associates with altered mucosal immune compartment's barrier function and/or inappropriate sequestration of the systemic immune compartment. Outcomes of the CDR under those circumstances are consistent with the recognized clinical heterogeneity and overlapping features of chronic inflammatory conditions.

Several poorly understood issues in immunology are addressed. The immunologic self is here defined as a constellation of molecular patterns that maintain a biologic system in equilibrium. It is unique to each individual and continuously changes as a result of physiologic and pathologic events. Furthermore, discussed is the importance of glycosylation and glycation processes. Also, emphasized is the essential role of innate mechanisms in immunity. In addition, addressed is the complex regulatory role of conventional effectors and their MHC-focused responses in maintenance of system's integrity in highly organized megaorganisms that require the specificity-driven TCR and BCR repertoire.

Finally, proposed are experimental approaches to test and validate the CDR concept of the immune system function.
